# The Interaction between Oral Bacteria and 3D Titanium Porous Surfaces Produced by Selective Laser Melting—A Narrative Review

**DOI:** 10.3390/biomimetics9080461

**Published:** 2024-07-29

**Authors:** Tatiane Cristina Dotta, Simonetta D’Ercole, Giovanna Iezzi, Vinicius Pedrazzi, Rodrigo Galo, Morena Petrini

**Affiliations:** 1 Department of Dental Materials and Prosthodontics, Ribeirão Preto School of Dentistry, University of São Paulo, São Paulo 14040-904, Brazil; tatianedotta@usp.br (T.C.D.); pedrazzi@forp.usp.br (V.P.); rogalo@forp.usp.br (R.G.); 2 Department of Medical, Oral and Biotechnological Sciences, University of Chieti-Pescara, 66100 Chieti, Italy; simonetta.dercole@unich.it (S.D.); gio.iezzi@unich.it (G.I.)

**Keywords:** selective laser melting, 3D titanium, surface, oral bacteria

## Abstract

The interaction between oral bacteria and dental implant surfaces is a critical factor in the success and longevity of dental implants. With advancements in additive manufacturing technologies, selective laser melting (SLM) has emerged as a prominent method for producing titanium implants with highly controlled microstructures and porosities. These 3D printed titanium surfaces offer significant benefits, such as enhanced osseointegration and improved mechanical properties. However, the same surface features that promote bone cell attachment and proliferation may also provide favorable conditions for bacterial adhesion and biofilm formation. Understanding the dynamics of these interactions is essential for developing implant surfaces that can effectively resist bacterial colonization while promoting tissue integration. This narrative review explores the complex interplay between oral bacteria and SLM-produced titanium porous surfaces, examining current research findings and potential strategies for optimizing implant design to mitigate the risks of infection and ensure successful clinical outcomes.

## 1. Introduction

Recent advances in medicine and dentistry have been significantly influenced by the evolution of techniques and materials, particularly in additive manufacturing. Among the various methodologies within this field, Selective Laser Melting (SLM) stands out as a particularly revolutionary technology [1,2,3]. Originating in the early 1980s, SLM has gradually gained prominence across a spectrum of scientific and industrial domains [4,5,6,7].

One of the most impactful applications of SLM technology is in dentistry, where it has revolutionized the fabrication of functional biomaterials, dental implants and prosthetic components. The capability of SLM to produce implants with intricate and personalized geometries represents a fundamental advantage of this technology [4,8,9,10,11]. Using a three-dimensional digital model, SLM employs a high-powered laser to selectively fuse thin layers of metallic powder, facilitating the creation of implants individualized for patients. This functional customization transcends traditional manufacturing methods and allows for precise adjustments in implant design details, including shape, size, and porosity [4,12].

Titanium, renowned for its unique properties such as high biocompatibility, corrosion resistance, and lightweight nature, is a material commonly employed in the fabrication of dental implants [13,14]. When combined with SLM technology, titanium enables the fabrication of highly complex and personalized pieces that were previously unfeasible. The precision and surface quality achieved through SLM enable the creation of implants with micrometer-scale details and intricate geometries, contributing to optimized osseointegration and adaptation to surrounding tissues [13,14].

A primary advantage of SLM in dentistry is its ability to produce controlled porous surfaces, a critical feature in medical applications such as bone implants [13,14]. These porous structures are designed to promote osteointegration and vascularization, facilitating more effective integration with surrounding tissue. Consequently, implants manufactured via SLM exhibit faster recovery and increased durability, thereby enhancing patient well-being and quality of life [13,14,15].

Despite the numerous benefits of SLM-produced implants, challenges remain to be addressed. The optimization of process parameters, such as temperature, scanning speed, and laser energy density, is essential to ensure the quality and structural integrity of manufactured parts. Additionally, validation of biocompatibility and regulatory compliance of final products are fundamental aspects, especially within the context of medical device manufacturing [16,17].

The interaction between oral bacteria and porous titanium surfaces is crucial in dentistry and medicine. These surfaces play a fundamental role in osseointegration and the effectiveness of biomedical implants. Porosity and surface roughness directly impact bacterial adhesion, colonization, and biofilm formation [10,18,19,20]. The porous structure offers a larger surface area for cell interaction, favoring cell migration and proliferation and consequently contributing to implant healing and stability [18,21]. On the other hand, porosity and increased roughness can create favorable sites for bacterial adhesion, increasing the risk of peri-implant infections and potentially leading to implant failure [10]. Although current, this topic remains underexplored in contemporary research and science. Therefore, understanding the interaction between bacteria and porous and rough titanium surfaces is crucial for the development of prevention and treatment strategies that ensure the safety and efficacy of dental implants and medical devices [10,18].

## 2. Selective Laser Melting (SLM) Technique

**Figure 1:**
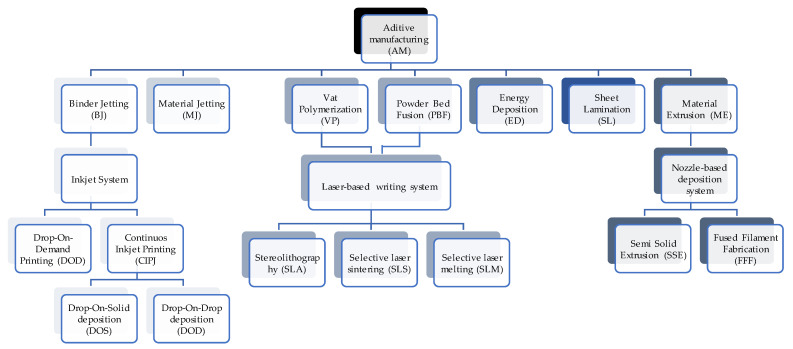
Additive manufacturing processes.

The most widely recognised classification for additive manufacturing methods is established by the ISO/ASTM 52900:2021 standard, which divides them into seven main classes: (1) Binder Jetting (BJ); (2) Material Jetting (MJ); (3) Vat Photopolymerization (VP); (4) Powder Bed Fusion (PBF); (5) Energy Deposition (ED); (6) Sheet Lamination (SL) and (7) Material Extrusion (ME) [1,22,23]. Figure 1 schematically exemplifies the additive manufacturing processes.

Among the emerging technologies that have revolutionised different industries, additive manufacturing stands out, with Selective Laser Melting (SLM) emerging as one of the most significant within this innovative field [3,9]. SLM represents a revolutionary breakthrough enabling the production of three-dimensional components with exceptional precision and unprecedented geometric complexity [8,18]. This technology utilizes precise digital models obtained through intraoral scanning or computerized tomography to create prosthetic structures directly from CAD (Computer-Aided Design) files [1,9,24]. The three-dimensional printing process allows for the creation of custom dental pieces, such as implants, crowns, and partial or complete prostheses, with micrometric precision [4,8,9,10,11,24].

According to a digital model, SLM employs a high-power laser to melt layers of metal powder (such as stainless steel, cobalt-chrome alloys, and titanium alloys), creating three-dimensional parts with high precision and geometric complexity. Each layer is melted and consolidated as the laser moves according to the CAD file data, ensuring proper fusion of the individual layers [2,24]. The thermal energy generated by the laser is sufficient to melt the thin layer of metal powder and fuse it with the underlying layers, creating a solid and continuous piece. As a result of this process, the final pieces feature compact internal structures, high dimensional accuracy, and excellent mechanical properties [1]. Technological advancements have facilitated the printing of layers with thicknesses ranging from 75 to 200 μm through the introduction of Selective Laser Melting machines equipped with multi-laser technology [4,12].

Among the critical parameters to be considered in printing are laser power, scanning speed, and energy density, which play a fundamental role in determining the quality and integrity of the manufactured parts [25,26]. Parts manufactured via SLM exhibit not only exceptional dimensional precision but also low surface roughness and impeccable structural integrity, highlighting the excellence of this technique in producing high-quality components at low cost [8,10,26].

A variety of materials can be processed by SLM, ranging from polymers and ceramics to metallic alloys [1,26]. Each of these materials presents specific characteristics when subjected to the SLM process, such as mechanical strength, biocompatibility, and thermal behavior, making them suitable for a wide range of applications [1,26].

Polymers processed by SLM provide a wide range of properties, ranging from flexibility to rigidity, depending on their composition, making them suitable for a variety of uses. This includes the manufacture of custom medical devices, engineering prototypes, and consumer parts [1,4].

Ceramics, when processed by SLM, exhibit remarkable thermal and chemical properties, making them ideal for high-temperature environments, such as in the wind energy, nuclear, and aerospace industries. Additionally, the ability to create controlled porous structures in porous ceramics by SLM opens doors for biomedical applications, such as bone implants, where osseointegration plays a crucial role [1].

On the other hand, metallic alloys processed by SLM often demonstrate exceptional mechanical and tensile strengths, making them ideal for applications requiring high durability and structural strength, including medical endoprosthesis of the hip and knee, pins, plates, screws, and dental implants [1,26]. Additionally, the ability to customize alloy composition enables the creation of materials tailored for specific applications, maximizing the performance of final components [1,26]. The materials used in this technique for the manufacture of metallic parts encompass a variety, including cobalt–chrome, carbon steel, stainless steel, aluminum, and titanium alloys [1].

Specifically, when processed by SLM, titanium offers significant advantages, including the creation of controlled porous surfaces that facilitate osseointegration in medical implants, promoting fusion with surrounding tissue and enhancing device biocompatibility [13]. Additionally, it is crucial to highlight that the porous nature of these structures provides an expanded surface area, favoring the growth and adherence of bone cells. This phenomenon facilitates bone incorporation into the material, endowing it with osteoconductive properties, i.e., the ability to support and stimulate bone tissue growth [13].

Titanium and its alloys enjoy a long and prominent history in dentistry due to their unique properties that make them ideal for a variety of dental applications [8,27], and these are recognized for the favorable combination of their mechanical, physical, and chemical properties, such as low density, high mechanical strength, high corrosion resistance and excellent biocompatibility [8,28,29].

One of the most prominent applications of titanium in dentistry is in the manufacture of dental implants [8]. Titanium implants are widely preferred due to their osseointegration capability, which allows the implant to integrate with the surrounding bone, providing a stable foundation for fixed or removable dental restorations. Additionally, the biocompatibility of titanium minimizes the risk of rejection by the body, making it a safe choice for a wide range of patients [13,30].

The surfaces of these alloys are deliberately designed with high porosity to optimize interaction with cells and stimulate rapid healing and bone integration [10,31]. As evidenced in the study by Zhang et al. (2018) [31], 3D titanium samples manufactured by SLM demonstrated excellent biocompatibility and the ability to promote the proliferation of dental pulp stem cells. These samples exhibited a surface resembling natural bone, facilitating cell adhesion and exhibiting osteoconductive properties, as indicated by the profile of osteogenic markers. These results emphasize the potential of porous titanium surfaces produced by SLM for application in dental implants, promoting a favorable biological response and facilitating integration with surrounding tissues.

While there are many types of titanium alloys, the Ti–6Al–4V alloy is the most commonly used due to a series of significant advantages [1,29]. The satisfactory biocompatibility of these alloys is an important advantage, as it allows for effective integration with surrounding tissue, reducing the risk of adverse reactions from the body to the implant. This is crucial for promoting a rapid and successful patient recovery [1,32,33].

However, its use has been questioned in recent years because its elastic modulus (110 GPa) is too high in respect to that of the bone tissue (10–30 GPa). This incompatibility can lead to “stress shielding”, resulting in aseptic implant failure due to bone atrophy and poor remodeling. Recent 3D printers have adopted various technologies for the manufacturing of metallic biomedical devices [9]. Among these techniques, the most notable ones are those utilizing powder bed fusion. In this process, a high-energy beam, such as a laser, is employed to perform Selective Laser Sintering (SLS) or SLM of successive layers of metal powder, deposited according to a CAD design model [1,2]. Additionally, the technique of Laser Direct Metal Deposition (LDMD) allows for controlled delivery of metal powder through a nozzle, enabling the production of structures with adjustable porosity and material gradients. The flexibility of the process enables the manufacturing of a wide range of parts with high precision, typically superior to 1 mm [27,34]. Furthermore, it is worth mentioning Electron Beam Melting (SEBM), an alternative to laser usage, which offers high production efficiency and minimizes part deformation during the manufacturing process [10,35].

## 3. Factors Influencing Bacterial Adhesion to Titanium

Despite its widespread acceptance in dentistry and other biomedical areas due to its notable biocompatibility and strength, titanium faces challenges also related to bacterial adhesion on its surface, which can result in serious complications [8,19,20,36]. Bacterial adhesion to titanium can trigger a range of issues, including local infections, inflammation of surrounding tissues, and even implant failure [8,13].

Understanding the mechanisms underlying bacterial adhesion and biofilm formation is essential for developing effective prevention and control strategies. Biofilms can be regarded as a multicellular growth phase in the bacterial life cycle [4,37]. Once bacterial cells adhere to a surface, they secrete an extracellular matrix composed of polysaccharides, proteins, and nucleic acids, which helps them stick together and form a cohesive structure. This matrix physically protects the bacteria and facilitates communication and resource sharing among the cells, developing complex, cooperative behaviors characteristic of a mature biofilm. Within this biofilm, bacterial cells can undergo significant phenotypic changes, including gene expression and metabolic activity alterations, which enhance their survival and adaptability [4].

One of the most challenging aspects of biofilms is their remarkable resistance to antibiotics and antimicrobial agents. This resistance can be up to 1000 times greater than that of free-floating, planktonic bacterial cells [4,38]. The reasons for this heightened resistance are multifaceted. The extracellular matrix acts as a barrier, limiting the penetration of antimicrobial substances. Additionally, the close proximity of cells within the biofilm facilitates the transfer of resistance genes. Moreover, the biofilm environment can trigger a stringent response—a stress response that redirects bacterial metabolism and slows down growth, making cells less susceptible to antibiotics that target actively dividing cells. This response, along with other changes in gene expression, enables biofilms to withstand hostile conditions, including the presence of antimicrobial agents [4,39].

The potential for adhesion between a bacterial cell and a substrate surface is influenced by several factors [40]. Among these, the bacteria’ physical and chemical properties, the substrate surface’s physicochemical characteristics, and the environmental conditions in which attachment occurs are prominent. These elements interact in a complex manner to determine the bacterial cell’s affinity for the surface, playing a crucial role in initial adhesion and subsequent bacterial colonization [40]. For example, the titanium surface’s topography, roughness, wettability, and chemical composition can significantly influence bacteria’s ability to adhere and form biofilms. Bacteria are usually negatively charged due to carboxyl, amino, and phosphate groups on their cell wall surfaces [41]; so, the interaction with positively charged surfaces is facilitated. Rougher surfaces tend to promote greater bacterial adhesion than smoother surfaces, and certain surface characteristics, such as microscopic irregularities or exposure to certain chemical groups, can facilitate or hinder bacterial adhesion [13]. Surface wettability is another property that can influence the cells’ interaction with titanium. Materials characterized by water contact angles < 90° are considered hydrophilic. Increasing the roughness of hydrophilic surfaces also increases wettability; on the other hand, by increasing the roughness of hydrophobic surfaces, the wettability decreases [42].

In addition to surface-related factors, other aspects, such as environmental and biological factors, can also influence bacterial adhesion to titanium. Environmental conditions, such as pH and humidity, can affect bacteria’s ability to adhere to the titanium surface. Similarly, biological factors, such as the presence of host proteins or cells, can modulate bacterial adhesion [13].

The oral cavity is home to many microorganisms, which play significant roles in oral conditions such as caries and periodontitis. For example, *Streptococcus mutans* and *Lactobacillus* spp. are recognized for their contribution to caries development, while *Actinobacillus actinomycetemcomitans*, *Porphyromonas gingivalis*, *Tannerella forsythia*, and *Treponema denticola* are associated with periodontitis [18,43]. As noted by Giulio et al. (2016) [18]. *P. gingivalis* emerges as the most predominant strain associated with advanced human periodontitis. This bacterium can colonize different types of host cells and secrete various virulence factors. It also establishes positive interactions with other oral colonizing microorganisms.

Furthermore, according to Chen et al. (2019) [28], *Staphylococcus aureus* and *Staphylococcus epidermidis* emerge as the main pathogens responsible for orthopedic device-related infections, with an estimated prevalence of peri-implantitis in about 22% of cases. According to D’Ercole et al. (2021) [10] and Hall et al. (2021) [4], *Streptococcus oralis* and *Streptococcus sanguinis* species, followed by *Neisseria pharyngis* and *Gemella haemolysans*, are considered the first colonizers of the implant surface.

These bacteria coexist in the oral cavity and depend on surface adhesion to survive and multiply. The ability to adhere and form biofilms on hard surfaces within the mouth is critical for developing and worsening oral diseases. Surface morphology and hydrophobicity are crucial in bacterial colonization capability and biofilm formation. Specifically, microscopic features such as valleys or depressions have been associated with a greater propensity for bacterial colonization. These concave structures provide favorable environments for bacterial adhesion and proliferation, promoting biofilm formation [10,44]. The interaction between *Streptococcus oralis* and different implant surfaces has been largely studied. Titanium sandblasting significantly increased the roughness of surfaces to an average of 100 nm of Ra, compared with machined surfaces that showed values of 25 nm [45]. Also, the wettability of sandblasted surfaces significantly increased. However, the sandblasting process was also correlated with an increase in the presence of oxygen concerning machined surfaces. The microbiological analysis showed no differences at 24 and 48 h for *Streptococcus oralis* CFUs and biofilm on the two surfaces. The authors hypothesized a fundamental antibacterial action due to the higher oxygen percentage in the sandblasted surfaces that counteracted their increase in roughness and hydrophilicity [46]. 

In another study, Petrini et al. [45] compared three different surfaces of the same manufacturer: machined, single-etched, and double-etched titanium (DAE). Double-etched discs showed a significant increase in porosity, hydrophilicity, superficial Oxygen, and nano-roughness that reached values of 200 nm. However, the higher roughness DAE surfaces were characterized by a significant decrease in *Streptococcus oralis* CFUs, and biofilm at 24 and 48 h.

D’Ercole et al. [47] analyzed the role of material in 2020, comparing microbiological interaction with titanium discs, machined and double-etched with peek samples. The authors showed that PEEK and machined were characterized by similar micro-roughness, but atomic force microscopy revealed that PEEK had a significantly higher nano-roughness. EDS analysis showed a significant increase in carbon and oxygen and lower wettability for peek samples. All these characteristics contribute to a reduction in the bacterial load in PEEK samples compared to machined and DAE samples.

## 4. Prevention and Treatment Strategies

### 4.1. Surface Topography and Porous Structure

The surface topography of implants produced by SLM plays a pivotal role in the intricate interaction between the implants and oral bacteria. This interaction can have substantial implications at various stages of the implant integration process, ranging from osseointegration to bacterial biofilm formation, directly impacting the stability and long-term success of the implanted devices [48,49].

Furthermore, contamination from residual powder and surfaces may occur during the manufacturing process of biomaterials, such as dental implants [8]. If not adequately removed, this contamination can accumulate in the implant’s surrounding tissues after insertion into the human body. This accumulation of contaminants can trigger a series of interactions between bone tissue cells and the immune system [50,51]. For example, osteoblasts, bone-forming cells, and macrophages, cells of the immune system, can be affected by the presence of these contaminants [50,52]. These interactions can trigger adverse inflammatory and immune responses in the implant’s local environment, thus compromising the osseointegration process, which is crucial for the long-term success of dental implants. Consequently, implant failure can occur due to these detrimental interactions between cells and residual contaminants [53,54,55,56].

Pore sizing is also crucial for porous structures intended for biomedical applications, especially to ensure successful osseointegration. A study by Karageorgiou et al. (2005) [57] highlighted that pores with a minimum diameter of 100 μm are necessary for proper cell migration. Gotz et al. (2004) [58] observed that a pore size of around 200 μm is particularly effective in promoting osseointegration in laser-textured Ti-6Al-4V implants. Furthermore, findings by Xue et al. (2007) [59] suggest that pores with a size larger than 200 μm are essential for facilitating cell growth within the pores. On the other hand, pores with diameters below 150 μm result in direct extension of cells through the pores without effective penetration. In contrast, Gallab et al. (2024) [13] introduced a novel titanium surface treatment that infused calcium and iodine ions into pores ranging from 900 μm to 300 μm. Initially releasing iodine ions to combat Staphylococcus aureus near the surface, larger pores posed a challenge with lower ion concentrations potentially diminishing effectiveness. Smaller pores, however, maintained higher ion levels, ensuring sustained antibacterial effects lasting over three months, thereby promoting optimal osseointegration of the implant with surrounding bone tissue.

Pattanayak et al. (2011) [60] proposed that Ti alloys with a porosity between 50 to 60% are more aligned with the mechanical properties of cortical bone. However, for higher porosity, above 70%, it is recognized as beneficial for bone growth; nevertheless, as porosity increases, the compressive strength of the implant tends to decrease [61,62]. Taniguchi et al. (2016) [63] conducted research indicating that Ti–6Al–4V implants with a pore size of 600 μm and a porosity of 64% exhibited superior fixation capability, fostering rapid bone growth and more favorable mechanical properties compared to those with pore sizes of 300 μm or 900 μm. Additionally, Chen et al. (2020) [64] observed that a pore size of 500 μm resulted in enhanced cell pre-seeding and osteogenesis compared to pore sizes of 600 μm and 700 μm. The impact of pore size and porosity on the performance of titanium implants is intricate and sometimes conflicting, posing challenges in achieving an optimal balance with a homogeneous structure. The ideal implant necessitates a harmonious blend of biological and mechanical attributes. In pursuit of meeting these criteria, graded porous structures have been devised, recognizing that natural bone itself comprises pores of diverse sizes [13].

### 4.2. Surface Roughness and Biofilm Formation

Surface roughness is a critical factor influencing bacterial adhesion and biofilm formation in biomedical materials [65]. According to Hu et al. (2008) [65], when the surface roughness exceeds 0.2 µm, it significantly enhances the likelihood of bacterial adhesion and subsequent biofilm development. This increased biofilm formation can elevate the risk of peri-implant inflammation and potentially compromise the long-term stability of implants. 

**Figure 2:**
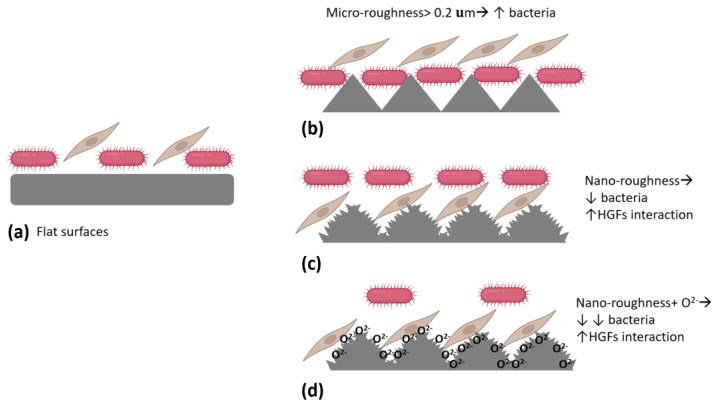
Bacterial adhesion and HGFs interaction based on surface roughness, in the first 48 h: (**a**) Flat surfaces, (**b**) surfaces characterized by micro-roughness > 0.2 µm present a higher bacterial accumulation with respect to flat surfaces; on the contrary, the presence of nano-roughness (**c**) is characterized by an increase in HGFs interactions and by a decrease in bacterial adhesion, (**d**) the presence of superficial oxidation increases the antibacterial actions of nano-rough surfaces, without affecting the HGFs interactions. Created with BioRender.com (accessed on 17 July 2024) and Microsoft^®^ PowerPoint^®^ for Microsoft 365 MSO (Version 2406).

Figure 2 clearly illustrates the mechanism of bacterial adhesion in relation to surface roughness. In Figure 2a, it is shown that smooth and flat surfaces pose a challenge for bacterial adhesion. In contrast, Figure 2b indicates that roughness greater than 0.2 µm leads to increased bacterial adhesion. Moreover, when surface roughness is at the nanoscale, there is a decrease in bacterial adhesion and an increase in the interaction with human gingival fibroblasts (HGFs), which contributes to maintaining oral health and preventing the development of periodontal diseases (Figure 2c).

Due to their layer-by-layer manufacturing process, Selective Laser Melting (SLM) samples inherently exhibit a range of surface roughness. This roughness varies widely, from 3.3 μm to 24 μm, depending on the specific machine and configuration parameters utilized. Such inherent surface roughness can create microenvironments that are conducive to bacterial colonization and biofilm formation [65,66,67].

Strategies to reduce surface roughness, such as polishing, can be explored to enhance clinical outcomes and mitigate complications associated with biofilm formation [44]. McGaffey et al. (2019) [44] conducted research addressing the impact of manual polishing on surfaces produced by 3D printing. Their results revealed a significant reduction in biofilm formation after this process. Moreover, they identified specific relationships between surface roughness and biofilm growth, indicating the importance of proper surface preparation. 

Additionally, various surface treatments can reduce the material’s roughness, creating an environment less conducive to bacterial proliferation. Longhitanoa et al. (2015) [68] investigated the surface roughness of Ti–6Al–4V alloys produced by the additive manufacturing technique of Direct Metal Laser Sintering (DMLS) under different surface treatments, including blasting, chemical etching, and electropolishing. The combination of blasting and chemical etching resulted in the lowest roughness. According to the authors, blasting creates a surface with uniform roughness by removing material due to the shape of the abrasive grains, which have cutting edges that remove material upon collision with the surface. Chemical etching, in turn, cleans the surface and reduces its roughness for two reasons: material removal occurs through surface oxidation, which ionizes the atoms that leave the matrix, and the complete immersion of the sample in the reagent allows all surfaces to be attacked. Conversely, electropolishing produced a mirror-like surface finish but with a high roughness value, demonstrating its ineffectiveness in reducing the surface roughness of the material.

These findings suggest that metallic implants manufactured through laser powder bed fusion may benefit from additional post-treatment procedures, like double etching. These additional steps can improve implant biocompatibility and reduce the likelihood of complications associated with biofilm formation.

### 4.3. Surface Treatment Methods

Effective and versatile methods have been applied to prevent infections associated with implants include implant surface treatment through techniques such as blasting and coating [21,65] as well as the incorporation of antimicrobial agents on the implant surface, such as silver-based agents and antibiotics [13,65].

The available techniques for coating three-dimensional porous titanium include various approaches, including immersion coating [69], sol-gel method [70], biomimetic [71,72,73], electrochemical [72,74,75], electrophoretic deposition (EPD) [76,77], electrochemical polishing (EL) and organic acids-etching (OAE) [78]. Each of these techniques has its own principles and specific processes. Immersion coating, for example, involves immersing the scaffold in a solution containing the desired coating material, followed by drying and curing [69]. The sol-gel method is based on the formation of a gel from chemical precursors, followed by heat treatment to produce the desired coating [70]. Biomimetic coating seeks to replicate the characteristics of the natural extracellular matrix [71,72,73], while electrochemical involves coating deposition through controlled electrochemical reactions [72,74,75]. Electrophoretic deposition (EPD) uses an electric field to deposit charged particles onto the porous scaffold surface, resulting in a uniform and adherent coating [76,77]. In the case of EL and OAE, the samples undergo a treatment to eliminate residual unmelted powder and microspheres that are loosely attached to the titanium surfaces [78].

Such approaches have the potential to significantly modify the implant surface morphology, thus influencing cell adhesion, proliferation, and differentiation. These interventions aim to create a surface less favorable for biofilm development and infections, thereby contributing to the prevention of postoperative complications and the long-term success of implant treatments [8,13]. Additionally, these modifications can influence the release of ions and bioactive molecules, which play an important role in regulating the biological response of bone tissue. Therefore, understanding how surface treatments impact the topography and biological properties of biomaterials is essential for the development of more effective and durable dental implants capable of promoting optimal osseointegration and reducing the risk of implant failure [79,80,81].

Di Giulio et al. (2016) [18] examined the biofilm formation of *Porphyromonas gingivalis* on grade 4 titanium (G4) discs and grade 5 Ti–6Al–4V alloy (G5) discs, with different surface treatments, including laser (L), sandblasting (S), and machining (M). The results indicated that the G4-L surface treatment was particularly effective in reducing biofilm formation, while the M treatment showed a significant increase in biofilm presence.

However, according to the study by Chen et al. (2023) [8], the addition of other components, such as copper (Cu), can benefit the microstructure and mechanical properties of SLM-produced TC4 alloy. The results revealed that adding 5% by weight of Cu caused changes in the alloy surface composition, increasing cell adhesion and proliferation of MC3T3-E1 cells. Additionally, an improvement in surface biological activity and a reduction in cytotoxicity levels were observed. Furthermore, applying physical surface modification methods, such as grinding and blasting, effectively removed powder residues and contaminants, resulting in reduced surface roughness and improved alloy biocompatibility.

Table 1 illustrates the effectiveness of various surface treatments applied to titanium produced via Selective Laser Melting in combating microorganisms.

Numerous researchers have successfully integrated antibacterial properties into 3D printing through the incorporation of silver ions (Ag) or silver nanoparticles (Ag NPs) [82,85,86]. Silver is well documented as a broad-spectrum antibacterial agent with a low propensity for inducing microbial resistance. However, its antibacterial effectiveness is typically limited to a duration of several weeks. For dental implants to achieve long-term success, it is crucial to maintain prolonged and stable antibacterial efficacy [65].

To address this requirement, constructing nano-scale structures on the surface of 3D printed materials present a promising approach. These nano-architectures can potentially provide sustained antibacterial properties by creating a surface that is inherently resistant to bacterial colonization and biofilm formation. This method leverages the unique characteristics of nanostructures to continuously inhibit bacterial growth, thereby enhancing the longevity and success of dental implants in clinical applications [65].

Van Hengel et al. (2017) [82] developed additively manufactured porous Ti–6Al–4V implants with antimicrobial functionality to prevent implant-associated infections, including those caused by methicillin-resistant *Staphylococcus aureus*. The proposed surfaces incorporated silver nanoparticles (AgNPs) into an oxide surface layer using Plasma Electrolytic Oxidation (PEO) in Ca/P-based electrolytes. The laser point size and layer thickness were 145 μm and 50 μm, respectively. Promising results demonstrated that AgNPs effectively inhibited biofilm formation, bacterial survival and growth in an ex vivo mouse femur infection model. According to the authors, PEO in the presence of AgNPs not only produces a bioactive surface with interconnected micro/nanoporosity, enhancing osseointegration but also dopes the implant surface with well-dispersed and firmly bonded AgNPs within minutes. Additionally, AgNPs are immobilized within a deep-growing oxide layer, preventing them from circulating freely in the bloodstream and avoiding potential nanotoxic effects. At the same time, the AgNPs are thoroughly dispersed within the extensive and hierarchical surface area of the additively manufactured porous implants, facilitating oxidation and the controlled release of Ag ions.

Macpherson et al. (2017) [83] also investigated the incorporation of Ag and Cu in their study, assessing their antimicrobial effects against Escherichia coli. In Ti–6Al–4V samples, 5 wt% of elemental Cu or 0.5 wt% of elemental Ag was introduced. The findings revealed that both +Cu and +Ag alloys demonstrated some degree of antibacterial activity, with measured antibacterial rates of 0%, 22%, 75%, and 99% for the Ti64, +Ag, +Cu, and 100% Cu samples, respectively. However, the +Cu alloy exhibited lower efficacy compared to similar alloys produced through casting methods. The authors attributed this discrepancy to the minimal formation of Ti_2_Cu due to rapid cooling during SLM processing, suggesting that appropriate heat treatment could enhance the antibacterial performance of the 3D printed material to match that of cast alloys. Conversely, the suboptimal antibacterial effectiveness of the +Ag sample indicated that the presence of Ag alone in the alloy did not adequately prevent biofilm formation by *E. coli*. Additionally, localized high concentrations of Cu or Ag might induce toxicity, while regions lacking these elements could compromise antibacterial efficacy.

In their 2018 study, Hu et al. [65] utilized sandblasting, anodization, and electrochemical deposition techniques to create a novel nanostructure of nanophase calcium phosphate (CaP) embedded within TiO_2_ nanotubes on micro-rough SLM titanium substrates (NTN). They compared these NTN samples with TiO_2_ nanotube (NT) samples, mechanically polished (MP) samples, and untreated SLM titanium samples. *Streptococcus mutans* and *Streptococcus sanguinis* exhibited significantly higher adhesion on the SLM samples than the NTN, NT, and MP samples, indicating that the nanostructured SLM titanium surfaces markedly reduced oral Streptococcus adhesion. The original surface roughness of the SLM specimens was 7.00 μm, much higher than the ‘Threshold Ra’ of 0.2 μm. Consequently, both live and dead *S. mutans* and *S. sanguinis* showed significantly higher adhesion than in the MP samples. Additionally, observations of dead bacteria with deformed and damaged membranes on the NTN sample surfaces under SEM suggested that the sharp edges of the CaP nanophase could penetrate and rupture bacterial cell membranes. These membrane-damaged bacteria might not firmly adhere to the NTN surfaces, explaining why dead bacteria adhesion did not significantly increase. Thus, the enhanced antibacterial properties of the NTN samples are primarily attributed to the increased nanoroughness and sharpness of the surface caused by the incorporated nanophase CaP. Furthermore, according to research by Ercan et al. (2011) [87], the combination of anodized titanium nanotubes and heat treatment reduced the adhesion of both live and dead Staphylococcus epidermidis and Staphylococcus aureus, particularly for nanotubes with a diameter of 80 nm, outperforming other treatment parameters. According to Campoccia et al. (2013) [88], smaller nanoparticles (especially those < 30 nm) and those with triangular or sharper shapes are likely to possess greater antibacterial properties.

The study conducted by D’Ercole et al. (2021) [10] compared topographical characteristics of different titanium-disk-manufacturing processes regarding antimicrobial outcomes. The evaluated groups included machined disks with grade IV titanium alloy, disks treated with double acid treatment (hydrofluoric and nitric acid) on grade IV titanium alloy, and TiAl6V4 disks with a 3D designed structure with an open-cell shape, with interconnected pores, produced by SLM. Remarkably, the 3D surface demonstrated superior effectiveness against *Streptococcus oralis* adhesion, especially when compared to machined disks. This discovery can be attributed to the characteristics of the new surface, including its nanoroughness, superficial oxygen presence, and exposed micro-surface area. [84]

In their research, Ji et al. [84] explored the combination of titanium (Ti) and copper (Cu) at varying weight concentrations of 3%, 5%, 7%, and 10%. The rapid solidification and cooling during the SLM process resulted in an average grain size reduction to 7.4 µm, significantly smaller than conventionally cast Ti, which typically exceeds 40 µm. This reduction in grain size was attributed to the presence of Cu-rich phase precipitates dispersed along the grain boundaries, which hindered grain growth. The Ti-3Cu alloy exhibited 99% antibacterial efficacy against *Escherichia coli*, while alloys containing 5%, 7%, and 10% Cu achieved nearly 100% efficacy, indicating robust antibacterial properties. The authors suggested that the structure of the new Ti-Cu alloy contributed to its remarkable antibacterial characteristics. Fine, unavoidable pores on the surface, resulting from shrinkage during rapid solidification, increased the actual contact area between bacteria and the alloy, enhancing the corrosion rate and facilitating the release of Cu^2+^ ions. Chloride ions in the solution could modify the permeability of the passive film, disrupting its structure and making the metal more prone to corrosion, thereby releasing Cu^2+^ ions. The potential mechanism underlying this antibacterial effect may involve Cu^2+^ ions extracting electrons from bacteria, compromising bacterial membrane permeability, leading to the loss of bacterial cytoplasm and the oxidation of the bacterial nucleus.

Contrary to what one might expect, where greater surface roughness could promote increased biofilm accumulation, Petrini et al. (2022) [78] observed the opposite. Samples of Ti–6Al–4V produced by SLM were subjected to electrochemical polishing (EL) and organic acid etching (OAE) to remove residual unmelted powder and microspheres loosely adhering to the titanium surfaces. The OAE-treated samples exhibited higher roughness at both nano and micro levels, with a wavy and inclined surface featuring very steep areas. However, they also demonstrated lower antibacterial and antibiofilm activity within 24 h against *Streptococcus oralis* than the EL-treated samples, which had a smooth, crack-free surface.

The heightened nano- and micro-roughness observed in the OAE samples arose from both organic etching and the SLM manufacturing process, involving fluctuations in high temperatures caused by the laser beam and subsequent cooling processes. Research suggests that surfaces treated with OAE exhibit robust interactions with soft tissues, particularly with collagen fibers oriented perpendicularly, forming a dense, intricate three-dimensional network extending across the surface in various directions. This treatment has been proposed for the production of abutments [78,89,90].

Therefore, while micro-asperities on titanium surfaces enhance surface–cell interactions, promoting osseointegration, they can also deter bacteria. Indeed, as shown by Lorenzetti et al. (2015) [91], the rigid structure of bacteria prevents them from adapting to the nano and micro-roughness of the surface, which acts as spacers between titanium and bacteria, thereby reducing bacterial adhesion.

Gallab et al. (2024) [13] devised an innovative surface treatment technique incorporating calcium and iodine ions onto titanium surfaces featuring pore diameters of 900, 600, and 300 μm. Additionally, they implemented a gradient structure with pore sizes transitioning from 900 μm externally to 600 and 300 μm internally. This treatment aimed to confer antibacterial properties against Staphylococcus aureus. The authors noted that halogen-based antibacterial compounds, such as iodine, typically operate through two mechanisms: kill-by-release and kill-by-contact. Initially, releasing iodine ions into the surrounding environment would eliminate bacteria near the titanium surface. If bacteria manage to penetrate this barrier and come into contact with the scaffolds, they are eradicated through direct contact.

Additionally, larger pore sizes can decrease concentration and limit the effective range of released iodine ions, thereby reducing the capacity to inhibit bacterial growth and survival near the scaffold. Consequently, the effectiveness of iodine ions in bacterial eradication primarily occurs at the scaffold’s surface, with diminishing antibacterial effects towards the center of the pore. Conversely, scaffolds with smaller pore sizes maintain a higher ion concentration within the pores, facilitating sustained long-term antibacterial effects lasting over three months. This duration is ample for ensuring complete osseointegration of the implant with the surrounding bone tissue [13].

Jiang et al. (2024) [33] employed PEO technology to modify the surface of the Ti–6Al–4V titanium alloy, incorporating bioactive strontium (Sr) and zinc (Zn) from calcium-phosphorus electrolytes. Their findings demonstrated that the bacterial colony count on the non-PEO titanium surface was approximately 290, whereas on the surface with the PEO film, the count decreased to about 140. The PEO film exhibited 52% antibacterial activity against *Staphylococcus aureus*, indicating that introducing Sr and Zn elements via PEO conferred antibacterial properties to the material. Topographical analysis revealed that these PEO films exhibited morphologies characterized by a typical microporous structure associated with PEO discharge, featuring volcano-like formations and localized microcracks. The surface of the film in the Z group displayed a distribution of submicron pores (<1 μm) and discharge pores (between 1 and 3 μm). The pore size continuously expanded with increased Sr concentration for the PEO films doped with Sr.

As per the same authors [33], porosity is influenced by two primary aspects. Firstly, a rise in process voltage can amplify micro-discharge, increasing the size and quantity of discharge pores and enhancing porosity. Secondly, an elevated process voltage may introduce additional molten oxide, potentially covering more discharge pores and reducing porosity. Additionally, a higher pH level increases the electrolyte’s alkalinity, leading to heightened arc discharge and oxide melting during the PEO process, thereby increasing the thickness and roughness of the coating. With a rise in the Sr^2+^ concentration in the electrolyte, there is a gradual increase in the thickness and roughness of the PEO coating. This can be attributed to the heightened process voltage and pH, which boost discharge intensity during the PEO process, accumulating more molten oxides on the titanium alloy surface, ultimately leading to a relatively thick and rough coating.

To address concerns regarding the Ti–6Al–4V alloys, such as its elastic modulus incompatibility and potential toxicities, Tardelli et al. (2024) [32] conducted a study comparing it with Ti–35Nb–7Zr–5Ta (TNZT), both produced through additive manufacturing. The study concluded that, although the TNZT alloy demonstrated higher hydrophilicity, surface free energy, and roughness, as well as lower hardness, there was no significant difference in colony-forming units (CFU) of *Staphylococcus aureus* when compared to Ti-6Al-4V. According to the systematic review by Tardelli et al. (2023) [92], roughness and moisture are two physical properties that significantly influence bacterial adhesion. Moreover, additive manufacturing techniques result in increased surface roughness. Primary stability shows rough implants offer a larger surface area, enhancing mechanical interlocking. However, for secondary stability, roughness can have a negative impact, depending on its influence on bacterial adhesion and bone cell attachment [32].

Moreover, it has been documented that ZnO nanostructures integrated into 3D printed materials demonstrate effective antibacterial activity owing to their distinctive surface properties [93,94]. Despite these promising attributes, the preparation process for these ZnO nanostructures is notably intricate and expensive. The complexity arises from the precise control required in the synthesis and deposition processes, which involve advanced techniques and equipment. Consequently, the cost and technical demands of producing these antibacterial ZnO nanostructures pose significant challenges to their widespread application in biomedical implants and devices [93,94].

Additive manufacturing and 3D printing are current topics that demonstrate great potential for enhancement and application. Understanding the mechanisms and factors involved in these techniques is crucial for seeking unresolved answers and improvement methods, ultimately aiming for success in rehabilitative treatments. Despite allowing for individualized construction and control of surface roughness, Selective Laser Melting (SLM) technology can still result in uncertainties and failures, such as bacterial adhesion on titanium alloys. The study by Palka et al. (2020) [95] evaluated the susceptibility to biofilm formation on Ti–6Al–4V alloys produced by SLM. Surface analysis revealed an average roughness of 102.75 nm and irregular topography of the tested plates, which were susceptible to biofilm formation by strains of *Streptococcus mutans*, *Staphylococcus epidermidis*, *Staphylococcus aureus*, *Lactobacillus rhamnosus* and *Candida albicans*. It is understood that the rough and irregular surface of biomaterials, especially their micro- and macrostructures, enhances osseointegration, a desirable process in implantology. Surface roughness and topography significantly correlate with bone regeneration and mechanical retention, but they also facilitate initial microbial adhesion and biofilm formation [95]. Therefore, as demonstrated in numerous studies, various treatments and modifications can be applied to material surfaces to prevent biofilm formation and bacterial adhesion.

In their comprehensive review, Sahm and colleagues (2024) [96] conducted a thorough examination of 543 publications, narrowing their focus to five selected for detailed investigation. Their findings indicated that enhancing titanium alloys with antimicrobial agents, whether through coating, modifying the alloy composition, or treating the surface, presents a viable and promising avenue. Importantly, these methods preserve the material’s mechanical integrity. 

However, it is evident that various physicochemical parameters of the implant, such as porosity, pore size, roughness, wettability, and chemical composition, strongly influence its bacterial behavior. Among these parameters, porosity and consequent surface roughness are widely considered to have a significant impact on bacterial adhesion and subsequent biofilm formation on titanium implants. Numerous studies have consistently shown that increased surface roughness enhances bacterial adhesion, whereas nanoscale roughness reduces bacterial adhesion and promotes interaction with human gingival fibroblasts (HGFs) [97,98,99,100,101]. This interaction supports better osseointegration and tissue health. Despite these findings, persistent limitations in the literature, such as substrate detachment and the duration of the antibacterial efficacy of nanomaterials, remain unresolved. Additionally, consensus on the most effective treatment approach has yet to be reached. Therefore, the anticipation lies in developing novel studies to address these existing knowledge gaps.

## 5. Conclusions

Bacterial adhesion to titanium surfaces depends on various physicochemical factors, including material composition, porosity, surface roughness, free energy, and wettability. This review underscores the critical role of 3D printed surfaces and post-production treatments in optimizing these factors. Post-production treatments, like organic acid etching, can improve the nano-roughness and modulate the superficial chemical composition of SLM titanium surfaces, enhancing the osteoblasts’ interaction and decreasing bacterial adhesion in the first 48 h.

## Figures and Tables

**Table 1:** Surface treatment of titanium manufactured by Selective Laser Melting (SLM) in combating microorganisms.

Author	Surface Treatment	Control Group	Pathogenic Species	Pre-Incubation of Samples in Saliva	Results
van Hengel et al., 2017 [82]	Silver nanoparticles in an oxide surface layer grown using Plasma Electrolytic Oxidation (PEO) in Ca/P-based electrolytes	Ti–6Al–4V	*S. aureus*	No	A decline in the quantity of CFU within an ex vivo infection model in mouse
Macpherson et al., 2017 [83]	Elemental addition of Ag or Cu	Ti–6Al–4V	*E. coli*	No	The Cu-containing alloy exhibited moderate antibacterial properties, outperforming the Ag-containing alloy
Hu et al., 2018 [64]	Sandblasting, anodization and electrochemical deposition of nanophase calcium phosphate (CaP)	TiO_2_	*S. mutans* and *S. sanguinis*	No	Reduction in the number of both types of bacteria in samples with surface treatment, compared to untreated TiO_2_ SLM
D’Ercole et al., 2021 [10]	Open cell form (interconnected pores)	Ti–6Al–4V	*S. oralis*	Yes	Three-dimensional discs exhibited considerably lower CFU levels and biofilm biomass than machined surfaces
Ji et al., 2021 [84]	Cu in varying concentrations (0, 3, 5, 7 and 10 wt%)	Ti	*E. coli*	No	The Ti-3Cu alloy showed 99% antibacterial efficacy against *E. coli*, while alloys with 5%, 7%, and 10% Cu achieved nearly 100% efficacy
Petrini et al., 2022 [78]	Electrochemical polishing (EL) and Organic acids-etching (OAE)	Ti–6Al–4V	*S. oralis*	Yes	OAE exhibited significantly lower CFU counts and biofilm biomass formation compared to EL and machined samples
Gallab et al., 2024 [13]	NaOH-CaCl2-heat-ICl3	Ti	*S. aureus*	No	Antimicrobial action lasting beyond three months, resulting in the complete elimination of bacteria
Jiang et al., 2024 [33]	Plasma Electrolytic Oxidation (PEO) in Zn and Sr-based electrolytes	Ti–6Al–4V	*S. aureus*	No	The PEO film demonstrated notable antibacterial characteristics
Tardelli et al., 2024 [32]	Ti–35Nb–7Zr–5Ta	Ti–6Al–4V	*S. aureus*	No	There was no difference in the CFU of bacteria among the groups
